# Association of gastrointestinal events and osteoporosis treatment initiation in newly diagnosed osteoporotic Israeli women

**DOI:** 10.1111/ijcp.12676

**Published:** 2015-08-17

**Authors:** J. Yu, I. Goldshtein, V. Shalev, G. Chodick, S. Ish‐Shalom, O. Sharon, A. Modi

**Affiliations:** ^1^ Merck & Co, Inc.KenilworthNJUSA; ^2^ Medical DivisionMaccabi Healthcare ServicesTel AvivIsrael; ^3^ Tel Aviv UniversityTel AvivIsrael; ^4^ Technion Faculty of MedicineHaifaIsrael; ^5^ Merck Sharp & Dohme Co. Ltd.Petah TikyaIsrael

## Abstract

**Background:**

The objective was to examine the association of gastrointestinal (GI) events and osteoporosis treatment initiation patterns among postmenopausal women following an osteoporosis diagnosis from an Israeli health plan.

**Methods:**

This retrospective analysis of claims records included women aged ≥ 55 years with ≥ 1 osteoporosis diagnosis (date of first diagnosis was index date). Osteoporosis treatment initiation was defined as use of osteoporosis therapy (oral bisphosphonates or other) during 12 months postindex. GI events (diagnosis of GI conditions) were reported for 12 months preindex and postindex (from index to treatment initiation or 1 year postindex, whichever occurred first). The association of postindex GI events (yes/no) with the initiation of osteoporosis treatment (yes/no) and with type of therapy initiated (oral bisphosphonate vs. other) were examined with logistic regression and Cox proportional hazard regression (as sensitivity analysis).

**Results:**

Among 30,788 eligible patients, 17.5% had preindex GI events and 13.0% had postindex GI events. About 70.6% of patients received no osteoporosis therapy within 1 year of diagnosis, 24.9% received oral bisphosphonates and 4.5% received other medications. Postindex GI events were associated with lower odds of osteoporosis medication initiation (85–86% reduced likelihood; p* *<* *0.01). Upon treatment initiation, postindex GI was not significantly associated with the type of osteoporosis therapy initiated, controlling for baseline GI events and patient characteristics.

**Conclusions:**

Among newly diagnosed osteoporotic women from a large Israeli health plan, 70.6% did not receive osteoporosis treatment within 1 year of diagnosis. The presence of GI events was associated with reduced likelihood of osteoporosis treatment initiation.

**What's known**

- Many women with osteoporosis do not initiate pharmacologic therapy to reduce their risk of fracture.
- Previous observational studies have linked gastrointestinal symptoms while on osteoporosis therapy with increased likelihood of therapy discontinuation but there is limited evidence examining the relationship between gastrointestinal events that occur following osteoporosis diagnosis and treatment initiation patterns.

**What's new**

- In this retrospective, claims‐based analysis of Israeli women newly diagnosed with osteoporosis, only 29% initiated osteoporosis treatment in the first year following diagnosis.
- Women with postdiagnosis gastrointestinal events were 85% less likely to initiate osteoporosis therapy than their counterparts who did not experience these events.
- Our results suggest that gastrointestinal events in women newly diagnosed with osteoporosis pose a significant barrier to osteoporosis treatment initiation.

## Introduction

Approximately, 200 million women are estimated to be diagnosed with osteoporosis worldwide 1, 2. The prevalence of osteoporosis among Israeli women, as diagnosed by a physician, has been reported to be approximately 14%, which is similar to the rate among women in the United States (US) 3. Menopause marks the beginning of an increased risk of osteoporosis as a result of the withdrawal of oestrogen, which accelerates the rate of bone remodelling, resulting in an imbalance between bone resorption and formation 4.

In a retrospective review of charts from 296 Israeli patients with osteoporosis‐related fractures, approximately two‐thirds of patients received no osteoporosis medication 5. Less than 5% of patients received bisphosphonates or hormone therapy; the rest received either calcium or vitamin D as osteoporosis treatment. In a summary of 17 other studies across several countries (Canada, US, Australia, and UK) measuring osteoporosis medication patterns among older patients with osteoporosis‐related fractures, Solomon and colleagues reported prescription osteoporosis treatment rates ranging from 1% to 51% 6, suggesting that, at best, half of older osteoporotic patients remain untreated.

Gastrointestinal (GI) events could be both a risk factor for osteoporosis development and an event occurring after initiating osteoporosis treatment. GI diseases, including inflammatory bowel disease and celiac disease, are known but often overlooked causes of osteoporosis 7. As a side effect of osteoporosis treatment, GI events are one of the most frequent causes of discontinuation of osteoporosis therapy. For instance, 2 European studies of patients taking bisphosphonates reported GI intolerance as the most common reason for treatment discontinuation 8, 9. In addition, a US longitudinal survey of osteoporotic women reported that women with GI side effects had higher osteoporosis treatment discontinuation rates, worse health‐related quality of life (HRQoL) scores, and lower treatment satisfaction scores than did women without GI side effects 10.

Furthermore, pre‐existing GI conditions appear to be risk factors for developing GI events after osteoporosis treatment 11, 12. This apparent association between pre‐existing GI conditions and subsequent osteoporosis treatment raises the question of whether these GI conditions limit the initiation of osteoporotic therapies. Although a number of studies 13, 14, 15, 16, 17, 18 have identified predictive factors for osteoporosis treatment initiation, few such factors have been reported consistently, except for the identification of bone mineral density testing as a positive predictive factor for treatment initiation 13, 15, 16, 18. Pre‐existing GI disease has rarely been tested for its predictive ability in osteoporosis treatment 14; thus, little is known about its association with osteoporosis treatment initiation.

The objective of this study was to examine the association of GI events and osteoporosis treatment initiation patterns, including whether treatment was initiated (yes/no) and, if yes, type of treatment initiated (oral bisphosphonates vs. other osteoporosis medications), among postmenopausal women following a diagnosis of osteoporosis from a large health plan.

## Methods

### Data source

Data for this analysis were obtained from the Maccabi Healthcare Services (MHS) database, which comprises the electronic medical records (EMR) of all patients from the MHS health maintenance organization (HMO) in Israel. The Maccabi database, which originated in 1998, contains records of approximately 2.7 million Israeli members, approximately 2 million of whom are active members.

### Study design

This was a retrospective analysis of the Maccabi database of claims from 1 January 2000 to 27 November 2012. The study population was defined using the following inclusion criteria: (i) any osteoporosis diagnosis (ICD‐9 733.0X), the date of which is referred to as the index date, (ii) female gender, (iii) ≥ 55 years of age at osteoporosis diagnosis and (iv) continuous enrolment in a Maccabi health plan beginning at least 1 year before and continuing at least 1 year after the index date. Maccabi participants were excluded if they met any of the following exclusion criteria: (i) oestrogen use during the year before the index date), (ii) Paget's disease of the bone (ICD‐9 731.0) diagnosed at any time in the claims history, and (iii) malignant neoplasm (ICD‐9 140–171, 173–208, 230–239) diagnosed 1 year before the index date through 1 year after the index date.

### Outcome variables

The two outcome variables of this study were osteoporosis treatment initiation during the post‐index period (yes/no) and the type of treatment initiated, specifically oral bisphosphonate medications (alendronate and risedronate) vs. other osteoporosis medications (denosumab, raloxifene, calcitonin, teriparatide and zoledronic acid). Ibandronate was not included as a treatment because it was not available in Israel at the time of the study.

### Other variables

Presence of pre‐index GI events and postindex GI events were reported in the study. Patients with pre‐index GI events were those with any GI procedure (Table S1) and/or GI diagnosis (Table S2) in the 1 year before the index date. Patients with postindex GI events were those with any GI procedure and/or GI diagnosis within the 12 months after the index date and before the initiation of any osteoporosis treatment, whichever occurred first.

The following data were extracted from the records of all eligible patients: (i) index date (date of osteoporosis diagnosis or osteoporosis fracture), (ii) age at index date, (iii) use of GI medication during the preindex period, if applicable [glucocorticoids, non‐steroidal anti‐inflammatory drugs [NSAIDs], gastro‐protective agents (proton pump inhibitors and H2 antagonists)], (iv) common osteoporosis‐related chronic comorbid conditions before index date (celiac disease, chronic inflammatory bowel, chronic inflammatory joint, depression, diabetes, fatigue, hyperparathyroidism, hypertension and renal failure), (v) non‐chronic comorbid conditions before the index date (urination problems and vitamin D deficiency) and (vi) Charlson comorbidity score at index date. Baseline characteristics were captured 1 year before the index date.

### Statistical analysis

Descriptive analysis was used for (i) the distribution of patients by the presence or absence of pre‐ and postindex GI events, (ii) the distribution of patients by the type of osteoporosis treatment (including no treatment), and (iii) the distribution of osteoporosis treatment by the presence or absence of pre‐ and postindex GI events.

Multivariate analysis was used to examine the relationship between the presence of postindex GI events (yes/no) and the initiation of any osteoporosis treatment (yes/no) and type of treatment initiated (oral bisphosphonate vs. other osteoporosis medication) among patients who initiated osteoporosis treatment. The multivariate analysis employed a logic regression model and included age, baseline GI medications and osteoporosis‐related comorbidities as adjustment variables. Sensitivity analyses were also performed. Because of the varying length of time among patients between index date and osteoporosis treatment initiation, the effect of postindex GI events on initiation of osteoporosis treatment (vs. no initiation of osteoporosis treatment) and on initiation of oral bisphosphonate treatment (vs. other osteoporosis medications) among those receiving any osteoporosis treatment was also examined by survival/“time to event” models, specifically by Cox proportional hazards regression using the same adjustment variables as the logistic regression model. Schoenfeld residuals test was used to assess the validity of the proportional hazards assumption.

## Results

### Patient sample and baseline characteristics

Of the 55,733 female patients with an osteoporosis diagnosis from 1 January 2000 through November 27, 2012, a total of 30,788 patients met all the eligibility criteria (Figure S1). The mean age [± standard deviation (SD)] of these patients was 65.0 ± 7.6 years; approximately one‐half (46.9%) of the patients were at least 65‐years old (Table 1). Approximately, 10% of the patient population used GI medications (gastro‐protective agents, 8.8%; NSAIDs, 1.3%; or glucocorticoids, 0.6%) during the preindex period. The most common chronic comorbidities present at the index date were hypertension (37.8%), chronic inflammatory joint (19.1%) and depression (16.1%).

**Table 1:** Baseline characteristics for patients included in the study

Baseline characteristics (1 year before the index date)	All patients *n* = 30,788
Age, mean (SD), years	65 (7.6)
**Age distribution, ** ***n*** **(%)**	
55–64 years	16,350 (53.1)
65–74 years	10,599 (34.4)
75–85 years	3,433 (11.2)
> 85 years	406 (1.3)
**Use of medications, ** ***n*** **(%)**	
Glucocorticoids	189 (0.6)
Prescription NSAIDs	398 (1.3)
Gastro‐protective agents	2719 (8.8)
Any GI event during preindex period, *n* (%)	5386 (17.5)
**Comorbid conditions, ** ***n*** **(%)**	
Hypertension	11,651 (37.8)
Chronic inflammatory joint	5885 (19.1)
Depression	4969 (16.1)
Diabetes	4055 (13.2)
Renal failure	1543 (5.0)
Fatigue	797 (2.6)
Chronic inflammatory bowel	727 (2.4)
Urination problems	685 (2.2)
Vitamin D deficiency	619 (2.0)
Hyperparathyroidism	298 (1.0)
Celiac disease	43 (0.1)
Charlson comorbidity index, mean (SD)^a^	2.1 (2.3)

a Among entire Maccabi population, score was 0.4 (1.3). GI, gastrointestinal; NSAID, nonsteroidal anti‐inflammatory drug; SD, standard deviation.

### Distribution of patients by the presence of GI events

Of the entire study population, 17.5% of patients had a preindex GI event; of these patients, 17.8% had a postindex GI event (before osteoporosis treatment initiation or end of follow‐up, whichever occurred first; Table 2). Overall, 13.0% of patients had a postindex GI event.

**Table 2:** Presence of gastrointestinal events

	Follow‐up period		Total
	Absence of postindex GI events	Presence of postindex GI events^a^	
**Baseline period**			
Absence of preindex GI events, *n* (%)	22,362 (88.0)	3040 (12.0)	25,402 (82.5)
Presence of preindex GI events, *n* (%)	4428 (82.2)	958 (17.8)	5386 (17.5)
Total, *n* (%)	26,790 (87.0)	3998 (13.0)	30,788 (100)

a Included those GI events collected prior to osteoporosis treatment initiation or within 1 year after index date, whichever came first. GI, gastrointestinal.

### Treatment initiation patterns

**Figure 1:**
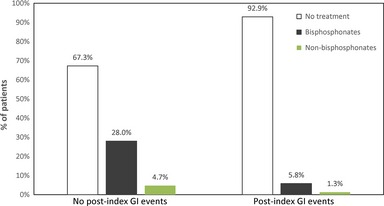
Osteoporosis treatment initiation by presence of postindex gastrointestinal event (*n* = 30,788)

Among the total study population (*n* = 30,788; Table 3), 70.6% of patients received no osteoporosis treatment in the year following their diagnosis. A similar proportion of patients was observed in a sensitivity analysis performed that included patients with 5 years of membership in the MHS HMO (rather than only 1 year). Additionally, 24.9% of patients received oral bisphosphonates, and 4.5% received other osteoporosis treatment; both groups of patients initiating therapy had a mean time to treatment initiation of less than 2 months. Of those 26,790 patients with no postindex GI events during the study period (Figure 1), 28.0% received oral bisphosphonate treatment and 4.7% received other osteoporosis treatment. A total of 5.8% of the 3,998 patients with postindex GI events during the study period received oral bisphosphonate treatment, whereas 1.3% received other osteoporosis medications.

**Table 3:** Treatment initiation (yes/no) and type of first osteoporosis therapy within 1 year of osteoporosis diagnosis

All patients *N* = 30,788	Treatment within 1 year of osteoporosis diagnosis	
	First osteoporotic therapy *n* (%)	Time to treatment initiation mean ± SD (months)
No treatment	21,744 (70.6)	N/A
Oral bisphosphonate	7670 (24.9)	1.5 ± 2.6
Other osteoporosis medications	1374 (4.5)	1.9 ± 2.9

N/A, not applicable; SD, standard deviation.

### Multivariate analysis results for treatment initiation

As shown in Table 4, patients with postindex GI events had lower odds of initiating any osteoporosis treatment, regardless of the presence or absence of preindex GI events. Among patients without preindex GI events, postindex GI events reduced the likelihood of initiating osteoporosis treatment by 85% (OR = 0.15; 95% CI, 0.13–0.17; p* *<* *0.01). Four risk factors for reduced likelihood of osteoporosis treatment initiation were identified in the population of patients without preindex GI events: age > 85, diabetes, depression and renal failure (p < 0.01). Within the same population of patients, age 65–74, age 75–85, baseline use of glucocorticoids or gastro‐protective agents, chronic inflammatory joint, hypertension, urination problems, hyperparathyroidism, vitamin D deficiency and fatigue were associated with greater odds of osteoporosis treatment initiation (p ≤ 0.04).

**Table 4:** Logistic regression analysis of postindex gastrointestinal events and osteoporosis treatment initiation

Independent variable	Among patients without preindex GI events				Among patients with preindex GI events			
	Odds ratio	95% CI		p value	Odds ratio	95% CI		p value
Presence of postindex GI event (ref: absence of post‐index GI event)	0.15	0.13	0.17	< 0.01	0.14	0.11	0.18	< 0.01
**Age at diagnosis (ref: 55–64 years)**								
65–74	1.42	1.34	1.52	< 0.01	1.33	1.16	1.52	< 0.01
75–85	1.28	1.17	1.41	< 0.01	1.12	0.91	1.38	.27
> 85	0.55	0.42	0.73	< 0.01	0.95	0.55	1.66	.87
**Preindex medication use**								
Gastro‐protective agents	1.29	1.15	1.44	< 0.01	1.31	1.12	1.53	< 0.01
NSAIDs	1.09	0.86	1.38	0.50	1.16	0.69	1.95	0.58
Glucocorticoids	2.07	1.46	2.94	< 0.01	2.28	1.24	4.18	0.01
**Comorbid conditions**								
Inflammatory bowel disease	1.18	0.98	1.43	0.08	1.05	0.74	1.48	0.78
Chronic inflammatory joint	1.09	1.02	1.17	0.02	0.96	0.82	1.11	0.56
Celiac disease	1.46	0.69	3.09	0.32	1.24	0.34	4.44	0.75
Diabetes	0.83	0.76	0.91	< 0.01	0.69	0.58	0.83	< 0.01
Depression	0.89	0.82	0.96	< 0.01	1.00	0.86	1.17	0.98
Renal failure	0.53	0.43	0.67	< 0.01	0.61	0.37	0.99	0.05
Hypertension	1.20	1.13	1.28	< 0.01	1.26	1.11	1.44	< 0.01
Urination problems	1.12	1.04	1.20	< 0.01	1.27	1.11	1.47	< 0.01
Hyperparathyroidism	1.32	1.01	1.72	0.04	1.52	0.84	2.75	0.17
Vitamin D deficiency	1.37	1.15	1.63	< 0.01	1.46	1.03	2.08	0.03
Fatigue	1.12	1.03	1.22	0.01	0.86	0.72	1.03	0.10

CI, confidence interval; GI, gastrointestinal; NSAIDs, nonsteroidal anti‐inflammatory drugs.

Among patients with preindex GI events, postindex GI events reduced the likelihood of initiating osteoporosis treatment by 86% (OR = 0.14; 95% CI, 0.11–0.18; p* *<* *0.01). Diabetes and renal failure were the only risk factors associated with a reduced likelihood of osteoporosis treatment initiation in the population of patients with preindex GI events (p ≤ 0.05). Within the same population of patients, age 65–74, baseline use of glucocorticoids or gastro‐protective agents, hypertension, urination problems and vitamin D deficiency were associated with greater odds of osteoporosis treatment initiation (p ≤ 0.03).

In the sensitivity analysis using Cox proportional hazards regression to account for varying time from osteoporosis diagnosis to treatment initiation, results were similar (data not shown in tables). Overall, patients with postindex diagnosis GI events were 83% less likely to initiate any osteoporosis treatment (hazard ratio = 0.17; 95% CI, 0.14–0.21; p* *<* *0.001). This analysis was performed with postindex GI events as a time‐varying covariate stratified to 4 time windows (< 90, 90–180, 181–270, 271–365 follow‐up days since index), because the hazard associated with this predictor was deemed non‐proportional with time.

Table 5 demonstrates that, within the population of patients receiving any osteoporosis treatment, the odds of initiating different types of therapy (oral bisphosphonate vs. other osteoporosis medications) was not significantly impacted by the presence of postindex GI events. Among patients without preindex GI events, postindex GI events reduced the likelihood of initiating oral bisphosphonate treatment by 23% (OR = 0.77; 95% CI, 0.54–1.10), which was not statistically significant (p* *= 0.15). Within the same population of patients, the baseline use of gastro‐protective agents, chronic inflammatory joint and fatigue were associated with reduced likelihood of using oral bisphosphonate medication (p < 0.01), while age of 65–74, age 75–85 and vitamin D deficiency were all associated with greater odds of oral bisphosphonate treatment initiation (p ≤ 0.03).

**Table 5:** Logistic regression analysis of postindex gastrointestinal events and type of treatment initiated (oral bisphosphonates over other osteoporosis medications)

Independent variable	Among patients without preindex GI events				Among patients with preindex GI events			
	Odds ratio	95% CI		p value	Odds ratio	95% CI		p value
Presence of postindex GI event (vs. absence)	0.77	0.54	1.10	.15	0.88	0.48	1.59	.66
**Age at diagnosis (ref: 55–64 years)**								
65–74	1.22	1.05	1.41	< 0.01	1.07	0.81	1.42	0.62
75–85	1.28	1.03	1.60	0.03	0.87	0.57	1.32	0.51
> 85	1.05	0.53	2.10	0.88	3.74	0.49	28.84	0.21
**Preindex medication use**								
Gastro‐protective agents	0.65	0.52	0.81	< 0.01	0.71	0.52	0.96	0.03
NSAIDs	0.77	0.47	1.25	0.29	1.17	0.39	3.46	0.78
Glucocorticoids	2.13	0.91	5.01	0.08	2.11	0.61	7.25	0.24
**Comorbid conditions**								
Inflammatory bowel disease	1.31	0.83	2.06	0.25	0.81	0.41	1.60	0.54
Chronic inflammatory joint	0.75	0.64	0.88	< 0.01	0.73	0.55	0.99	0.04
Celiac disease	2.02	0.26	15.71	0.50	0.53	0.05	5.16	0.58
Diabetes	1.22	0.99	1.52	0.07	1.13	0.75	1.70	0.57
Depression	1.00	0.84	1.21	0.96	1.00	0.72	1.37	0.98
Renal failure	0.83	0.48	1.43	0.50	0.53	0.20	1.40	0.20
Hypertension	1.13	0.98	1.31	0.08	1.36	1.03	1.79	0.03
Urination problems	0.93	0.60	1.44	0.75	0.73	0.39	1.36	0.32
Hyperparathyroidism	1.20	0.63	2.27	0.58	–a	N/A	N/A	N/A
Vitamin D deficiency	2.11	1.24	3.59	< 0.01	1.70	0.66	4.41	.27
Fatigue	0.56	0.40	0.79	< 0.01	0.38	0.19	0.73	< .01

a The small number of cases of hyperparathyroidism among treated patients leads to quasi complete separation (no observations of patients with hyperparathyroidism without postindex GI events among patients with preindex GI events). This resulted in very large maximum likelihood estimates. CI, confidence interval; GI, gastrointestinal; N/A, not applicable; NSAIDs, nonsteroidal anti‐inflammatory drugs.

Among patients with preindex GI events, postindex GI events reduced the likelihood of initiating oral bisphosphonate by 12% (OR = 0.88; 95% CI, 0.48–1.59), which was not statistically significant (p* *= 0.66). Within the population of patients with preindex GI events, the baseline use of gastro‐protective agents and the baseline comorbidities of chronic inflammatory joint and fatigue were risk factors for reduced likelihood of oral bisphosphonate treatment initiation (p ≤ 0.04), whereas hypertension was associated with greater odds of oral bisphosphonate treatment initiation (p = 0.03). In the sensitivity analysis using Cox proportional hazards regression to account for varying time from osteoporosis diagnosis to oral bisphosphonate treatment initiation, results were similar (data not shown in tables).

## Discussion

Overall, 17.5% of patients had GI events in the year prior to osteoporosis diagnosis (index date) and 13.0% had GI events after this date (but before initiation of any osteoporosis therapy). Approximately, 70% of patients did not initiate any pharmacological treatment for osteoporosis within one year of being diagnosed with osteoporosis. Postindex GI events reduced the odds of any osteoporosis treatment initiation by approximately 85%, regardless of the presence or absence of GI events prior to osteoporosis diagnosis or fracture, and sensitivity analyses confirmed these results. Among those patients who initiated any osteoporosis treatment, postindex GI events were not associated with type of medication used (oral bisphosphonate vs. other osteoporosis medications); this was true regardless of the presence or absence of preindex GI events.

The treatment initiation rate of 29.4% in this study is similar to other studies of women with a National Osteoporosis Foundation (NOF) indication for osteoporosis treatment (without information on fractures), which reported prescription osteoporosis treatment initiation rates (not including calcium and/or vitamin D) of 24–42% 13, 17, 19, 20, 21. The initiation rate in this study was higher when compared with studies examining the prefracture osteoporosis treatment rate among those patients presenting with a fracture. These rates ranged from 8% to 17% 22, 23, 24. Even after fractures, patients reported in the literature tend to have low osteoporosis treatment initiation rates, ranging primarily from 8% to 24% 13, 23, 25, 26, 27, 28, 29, 30.

This study examined the association of GI events and osteoporosis treatment initiation and found that the occurrence of postindex GI events was associated with a lower likelihood of osteoporosis treatment initiation. However, it did not signal significant interaction with the type of osteoporosis treatment initiated (oral bisphosphonate medications vs. other osteoporosis medications).

As with any retrospective analysis, this study has several limitations resulting from its study design. As reasons for not initiating osteoporosis treatment following diagnosis or fracture were not available in the claims database, it is not possible to draw firm conclusions regarding the reasons behind the initiation rate differences in patients with the absence or presence of postindex GI events. Furthermore, the severity of the GI events was not examined, so it cannot be determined whether this factor impacted osteoporosis treatment initiation rates. This study utilised age ≥ 55 as a surrogate for postmenopausal status; this is commonly used in osteoporosis studies 31, 32, but it is an imperfect surrogate. However, > 90% of women by age 55 are menopausal or postmenopausal, and by age 60, nearly 100% of women are postmenopausal 33. Therefore, this would suggest that less than 3% of women in our current study were premenopausal, so the use of this surrogate likely had little impact on the results. Finally, the results of this analysis may not be applicable to osteoporotic patients younger than 55, as they were not included in this patient population.

Among older women who were newly diagnosed with osteoporosis, less than 30% initiated treatment within 1 year of diagnosis, highlighting the need to improve the management of osteoporotic patients. The presence of a GI event following diagnosis was associated with a reduced likelihood of osteoporosis treatment initiation, suggesting the need for novel therapies with a favourable GI tolerance profile.

## Author contributions

GC: concept/design, data interpretation and approval of article. IG: concept/design, data analysis, data interpretation and approval of article. AM: concept/design and approval of article. OS, VS and SS: data interpretation and approval of article. JY: concept/design, data interpretation, critical revision of article and approval of article.

## Supporting information


**Table S1.** Gastrointestinal procedure codes used to define gastrointestinal event.
**Table S2.** Gastrointestinal diagnoses used to define gastrointestinal event.
**Figure S1.** Patient inclusion/exclusion steps.
