# Supplementary material for: Association of gastrointestinal events and osteoporosis treatment initiation in newly diagnosed osteoporotic Israeli women
Source: Int J Clin Pract. 2015 Aug 17;69(9):1007–14. doi: 10.1111/ijcp.12676 (PMC5042045; doi:10.1111/ijcp.12676)
Supplement: Supplementary file 1 — Table S1. Gastrointestinal procedure codes used to define gastrointestinal event. Table S2. Gastrointestinal diagnoses used to define gastrointestinal event. Figure S1. Patient inclusion/exclusion steps. [file IJCP-69-1007-s001.docx]

**Supplementary Materials (Online Resource)**

Association of Gastrointestinal Events and Osteoporosis Treatment Initiation in Newly Diagnosed Osteoporotic Israeli Women

Jingbo Yu, PhD^1^ (corresponding author)

Email: jingbo.yu@merck.com

Inbal Goldshtein, MSc^2^

Varda Shalev, MD, MPA^2, 3^

Gabriel Chodick, MS, PhD^2, 3^

Sophia Ish-Shalom^4^

Ofer Sharon, MD^5^

Ankita Modi, PhD^1^

^1^ Merck & Co, Inc. 2000 Galloping Hill Rd, Kenilworth, NJ, 07033 USA

^2^ Medical Division, Maccabi Healthcare Services, 27 Ha’Mered Street, Tel Aviv, Israel

^3^ Sackler Faculty of Medicine, Tel Aviv University, Haim Levanon St 55, 6997801, IsraelTel Aviv, Israel

^4^ Technion Faculty of Medicine, Efron St. no number, Haifa 31096, Israel

^5^ Merck Sharp & Dohme Co. Ltd., 32 Ha’Shacham St., P.O.B. 7121, Petah Tikya 49170, Israel

**Table 1. GastrointestinaI procedure codes used to define gastrointestinal event**

| **ICD-9-CM** | **Description** |
| --- | --- |
| 742200000 | X-ray - esophagus |
| 742400001 | X-ray and/or cineradiography, esophagus, stomach and duodenum with or without delayed films |
| 742450002 | X- ray small bowel follow-through study |
| 743600001 | Intraluminal esophageal strictures / obstructions dilat + rs dilation of strictures and/or obstructions - |
| 432000001 | Esophagoscopy, rigid or flexible, diagnostic with or without collecting of specimens, brushing or washing |
| 432340001 | Gastroscopy at Maccabi |
| 432340005 | Duodenoscopy |
| 432350000 | Upper gastrointestinal endoscopy, with or without collection of specimen (gastroscopy) |
| 432390001 | Biopsy - peroral gastro / intestinal |
| 432490000 | Dilatation of esophagus during upper gastrointestinal endoscopy |
| 434500001 | Dilation of esophagus without radiologic guidance |
| 434560002 | Esophageal dilation and insertion of stent |

**Table 2. Gastrointestinal diagnoses used to define gastrointestinal event**

| **ICD-9-CM** | **Description** |
| --- | --- |
| 456 | Esophageal varices+hemorrhage |
| 456.1 | Esophageal varices no hemorrhage |
| 530 | Esophagus achalasia |
| 530 | Achalasia & cardiospasm |
| 530 | Esophagus achalasia |
| 530.1 | Esophagitis |
| 530.11 | Esophagitis reflux |
| 530.19 | Eosonophilic esophagitis |
| 530.2 | Barrett's esophageal synd |
| 530.2 | Peptic ulcer esophagus |
| 530.2 | Peptic ulcer esophagus |
| 530.3 | Esophageal obs |
| 530.3 | Esophagus stricture |
| 530.3 | Schatzki's ring (esophagus) acquired |
| 530.4 | Esophagus perforation |
| 530.5 | Esophageal spasm |
| 530.5 | Esophageal spasm |
| 530.6 | Diverticulum esophagus |
| 530.6 | Zenker's diverticulum |
| 530.6 | Diverticulitis/osis esophagus acquired |
| 530.7 | Mallory-weiss synd |
| 530.81 | Esophageal reflux |
| 530.81 | Laryngopharyngeal reflux disease |
| 530.9 | Esophagus dis uns |
| 530.9 | Esophagus dis uns |
| 531 | Gastric ulcer acute+hemorrhage |
| 531.3 | Gastric ulcer acute |
| 531.4 | Gastric ulcer+hemorrhage |
| 531.7 | Gastric ulcer chronic |
| 531.9 | Gastric ulcer |
| 531.9 | Gastric ulcer no hemorrhage |
| 531.9 | Peptic ulcer gastric |
| 531.9 | Ulcer pyloric |
| 532 | Duodenal ulcer acute+hemorrhage |
| 532.7 | Duodenal ulcer chronic |
| 532.9 | Duodenal ulcer |
| 532.9 | Peptic ulcer duodenal |
| 533 | Peptic ulcer site uns acute |
| 533 | Peptic ulcer site uns acute+hemorrhage |
| 533.00 | Peptic disease |
| 533.9 | Peptic ulcer uns |
| 533.9 | Stress ulcer |
| 534 | Gastrojejunal ulcer+hemorrhage |
| 534.9 | Gastrojejunal ulcer uns |
| 537.4 | Gastrocolic fistula |
| 537.4 | Gastrojejunocolic fistula |
| 537.81 | Pylorospasm |
| 537.89 | Duodenum deformed bulb |
| 537.9 | Gastropathy nsai drug induced |
| 537.9 | Stomach & duodenum dis uns |
| 537.9 | Stomach & duodenum dis uns |
| 569.83 | Perforation of intestine |
| 578 | Hematemesis |
| 578 | Hematemesis |
| 578.1 | Bleeding stools |
| 578.1 | Melena |
| 578.9 | G-i hemorrhage uns |
| 578.9 | Gastrointestinal bleeding-upper gi tract |
| 578.9 | Gastrointestinal bleeding-lower gi tract |
| 578.9 | Gastrointestinal bleeding-upper gi tract |
| 787 | Nausea and vomiting |
| 787 | Nausea and vomiting-oncologic patient |
| 787.02 | Nausea |
| 787.03 | Emesis |
| 787.03 | Vomiting physiological |
| 787.1 | Heartburn |
| 787.2 | Dysphagia |
| 787.2 | Odynophagia |
| 789 | Abdominal pain |
| 789 | Cramps abdomen |
| 789 | Colic infantile |
| 789 | Flank pain |
| 789 | Groin pain |
| 789 | Abdominal pain - recurrent |
| 789 | Pelvic (abdominal) pain - male |
| 789.03 | Lower abdominal pain |
| 789.06 | Epigastric distress |
| 792.1 | Stool occult blood |
| 793.4 | Abnor imaging study g-i tract |

**Figure 1. Patient inclusion/exclusion steps**

Female patients ≥ 55 years old with an osteoporosis diagnosis

N = 55,733

Excluded patients who had taken osteoporosis medication during the data collection period prior to osteoporosis diagnosis

n = 10,616

Patients naïve to osteoporosis medication

n = 45,117 (81.0%)

Excluded patients not continuously enrolled in Maccabi health plan for at least 1 year ± osteoporosis diagnosis

n = 6,246

Excluded patients with estrogen use 1 year prior to osteoporosis diagnosis

n = 4,110

Patients continuously enrolled in Maccabi health plan for at least 1 year ± osteoporosis diagnosis

n = 38,871 (69.7%)

Excluded patients with diagnosis of malignant neoplasm or Paget’s disease of the bone

n = 3,973

Patients without malignant neoplasm or Paget’s disease of the bone

n = 34,898 (62.6%)

Patients without estrogen use 1 year prior to osteoporosis diagnosis

n = 30,788 (55.2%)
